# New Advances in Membrane Separation Technology for Water Pollution Control and Membrane Fouling Mitigation

**DOI:** 10.3390/membranes16060199

**Published:** 2026-06-08

**Authors:** Yilin Fan, Zhonglong Yin

**Affiliations:** Jiangsu Key Laboratory of New Power Batteries, Jiangsu Collaborative Innovation Center of Biomedical Functional Materials, Jiangsu Key Laboratory of Micro Nano Sensing and Separation Science for Analytical Chemistry, School of Chemistry and Materials Science, Nanjing Normal University, Nanjing 210023, China

Membrane separation technology, with advantages including high treatment efficiency, small footprint and easy operation, is playing an increasingly significant role in processes such as desalination, industrial separation, zero-liquid discharge (ZLD), water remediation, wastewater treatment and reclamation, and resource recovery [1,2]. However, fouling is “the Achilles heel” of membrane technology, causing flux loss and reducing lifetime, which in turn largely restrict its applications in water pollution control [3].

Common cleaning practices in practical membrane processes include feed water pretreatment (e.g., coagulation, adsorption, and oxidation) and chemical cleaning using reagents (e.g., acid and base); however, these methods feature drawbacks such as intensive chemical consumption and long duration [4]. Consequently, it is imperative to develop more effective and sustainable methods of controlling water pollution, such as antifouling membrane materials and modules, optimizing membrane processes, or combining membranes with other processes (e.g., catalysis) [5,6,7].

In this Special Issue, we focus on advancements in membrane separation technology which aim to control water pollution and mitigate membrane fouling. Research areas included, but were not limited to, membranes for ion/molecule separation, separation technology for drinking water/wastewater pollution control, antifouling membrane materials, membrane cleaning and advanced fouling control strategies, self-cleaning membranes, and catalytic membranes for enhanced separation and fouling control.

Nine papers are included in this Special Issue, encompassing recent developments in the fields of membrane materials, feed spacer design, and the prediction and mechanisms of fouling. Advanced membrane materials and modules are vital for controlling fouling: for example, Kasongo et al. (Contribution 1) grafted 3-allyl-5,5-dimethylhydantoin to thin-film composite (TFC) reverse osmosis (RO) membranes to improve their biofouling resistance against bacteria (*E. coli* and *S. aureus*). Yin et al. (Contribution 2) reviewed the materials used in electrocatalytic self-cleaning membranes for sustainable fouling control, including carbonaceous materials, metals and metal oxides, metal–organic frameworks (MOFs), covalent–organic frameworks (COFs), and MXene. In addition, Wang et al. (Contribution 3) designed a novel feed spacer for spiral-wound membranes based on simulations (computational fluid dynamics and discrete phase model) and experiments (optical coherence tomography and confocal laser scanning microscopy), which achieved lower fouling resistance and energy consumption (76%).

In-depth insights into the mechanisms and performance predictions of fouled membranes are critical for fouling control. Chen et al. (Contribution 4) clarified the ultrafiltration membrane fouling mechanisms in 1,3-propanediol fermentation broths and identified the main foulants (proteinaceous substances). Furthermore, Lakner et al. (Contribution 5) predicted expected fouling time during transmembrane transitions in RO systems using a probability-based model. Bahig et al. (Contribution 6) evaluated the NF fouling control performance of slow sand and zeolite hybrid pretreatment systems. The results demonstrated that proper pretreatments could decrease the turbidity, iron, and organic load in feed water, leading to lower fouling propensity.

Additionally, Mahlangu et al. (Contribution 7) investigated the influence of salt rejection properties on concentration polarization effects during colloidal fouling. They observed that NF90 membranes achieved a higher NaCl rejection rate (>97% vs. 46%) and flux decline (30–66% vs. 10–55%) compared to NF270 membranes, owing to cake-enhanced concentration polarization. Pervov et al. (Contribution 8) utilized low-rejection nanofiltration membranes to reduce concentration and achieve salt–organic separation during landfill leachate treatment, demonstrating a lower energy consumption compared to a high-pressure RO membrane. Lastly, Abdul Rahiman et al. (Contribution 9) reviewed the use of combined anaerobic ammonium oxidation (anammox)–membrane bioreactor (MBR) technology for nitrogen removal from wastewater.

This Special Issue presents state-of-the-art studies on various approaches to water pollution control and membrane fouling mitigation, which may provide guidance for fabricating advanced membrane materials and modulating processes. We also hope that this Special Issue inspires further research in this field.
